# Older Seniors during the COVID-19 Pandemic—Social Support and Oral Health-Related Quality of Life

**DOI:** 10.3390/healthcare9091177

**Published:** 2021-09-07

**Authors:** Sophia Weber, Sebastian Hahnel, Ina Nitschke, Oliver Schierz, Angelika Rauch

**Affiliations:** 1 Department of Prosthetic Dentistry and Materials Science, University of Leipzig, 04103 Leipzig, Germany; sebastian.hahnel@medizin.uni-leipzig.de (S.H.); ina.nitschke@medizin.uni-leipzig.de (I.N.); oliver.schierz@medizin.uni-leipzig.de (O.S.); angelika.rauch@medizin.uni-leipzig.de (A.R.); 2 Clinic of General, Special Care and Geriatric Dentistry, University of Zurich, 8032 Zurich, Switzerland

**Keywords:** COVID-19, ENRICHD social support inventory, oral health impact profile, oral health-related quality of life, older adults, older seniors, patient-reported outcome measure, PROM, social support

## Abstract

The coronavirus disease (COVID-19) has greatly affected all parts of private life and led to social distancing and self-isolation. Limited social support for older or frail people might have led to decreased oral health and its related quality of life. The current study aimed to investigate the social support of older seniors and self-perceived oral health-related quality of life (OHRQoL) over the course of the COVID-19 pandemic. Questionnaires were sent to all patients of the Dental Clinic of the University of Leipzig (Germany) aged 75, 80, or 85 years (*n* = 1228) at the end of February 2021. Besides demographic characteristics and care level, an adapted German version of the ENRICHD Social Support Inventory (ESSI-D) and the German Oral Health Impact Profile-14 (OHIP-14) were included. The response rate was 35.7% (*n* = 439). Twelve replies were not included in the data analysis as participants had either no interest, were cognitively impaired, or did not match the required age group. Analysis of ESSI-D revealed low social support for 13.2% (*n* = 53/403) of the seniors. No statistically significant impact of assigned care level on low social support was identified. Seniors with an assigned care level (CL_yes_) presented higher OHIP-14 sum scores (CL_no_/CL_yes_ 6.43/10.12; *p* < 0.001). This was also true for six of the seven OHIP-14 domains, except for functional limitation. Regarding seniors with an assigned care level, a weak positive correlation was identified for sum scores of the ESSI-D and the OHIP-14 (*r* = 0.29). Despite the COVID-19 pandemic, older seniors reported high self-perceived social support. As seniors with an assigned care level revealed more impaired OHRQoL and a correlation with lower social support, special attention should be given to this vulnerable and frail group in times of a pandemic. When restrictions may minimize access to dental treatment and might negatively influence the oral health of older seniors, health care programs should offer more individual options for dental/medical appointments.

## 1. Introduction

For more than a year, the coronavirus disease (COVID-19) has been spreading around the globe. What started as a “public health emergency of international concern” [1] was announced a pandemic shortly thereafter [2]. As the transmission of the virus is vastly progressing, the pandemic is having a major impact on different areas, such as economics, globalization, the health system, and people’s private lives. The latter is particularly influenced by the policy of social distancing [3], an important tool for controlling the spreading of the virus. Especially in times when vaccines are not available and incidence is high, minimizing physical contact with other people serves as a key instrument for a delay or flattening of the epidemic curve [4]. Nonetheless, several studies reported that loneliness might result in psychological distress, e.g., anxiety or depression [5,6,7]. Moreover, loneliness [8] and a lack of social support [9] coincide with decreased self-perceived well-being [8] and lower health-related quality of life [10], while the risk of frailty [11] and mortality [12] increase accordingly. Due to individual losses later in life, such as the death of a partner or close friends, loneliness commonly increases with older age [13], leading to a smaller social network and, therefore, a potential reduction in social support. Moreover, with increasing age, people tend to estimate their self-perceived social support as lower than before [14].

Older people and those presenting co-morbidities such as cardiovascular diseases, diabetes mellitus, or diseases affecting the respiratory system are at high risk for a COVID-19 infection [15]. Especially in dental practices, where clinical treatments involve the production of droplets and aerosols, a possible risk for the transmission of the coronavirus has been discussed [16,17], which has also led to concerns in the patient population [18]. In addition, crowded waiting rooms in medical institutions might produce a risk of spreading the virus among patients [19]. Consequently, frail or high-risk patients are likely to postpone or even cancel medical appointments [20], which possibly causes an impairment of their health status. Structural changes in hospitals or dental/medical practices during the pandemic made it more difficult for “non-emergency patients” to make appointments, especially when opening hours of ambulances were reduced in favor of supporting intensive care units. Therefore, it might have been essential that seniors were embedded into a supportive social environment that helped to maintain preventive health measures or medical therapies. This is especially true for older people depending on caregivers. As part of German statutory health insurances, this vulnerable group is monetarily supported by nursing care insurance [21]. With the help of a structured assessment, the grade of impairment is determined and used for the assignment of a care level (CL), with higher levels (CL1–CL5) representing an increasing impairment of mobility, cognitive and psychological function, independent lifestyle, disease-related burdens or social life, which finally results in a higher amount of support required [22]. In the assessment, an expert evaluates up to 16 criteria in each category, adding up to a maximum sum score of 100 points. While CL1 (sum score 12.5 ≤ 27) represents a minor impairment of independence, the impairment can be more severe (CL2: sum score 27 ≤ 47.5, significant impairment; CL3: sum score 47.5 ≤ 70, severe impairment; CL4: sum score 70 ≤ 90, most severe impairment; CL5: sum score 90–100, most severe impairment with special requirements for nursing care). Numerous long-term care facilities experienced severe COVID-19 outbreaks and became hotspots for the infection of residents and staff [23,24], which caused massive restrictions in social support resulting from isolation measures and a high workload for staff. In terms of oral health, older seniors (75–100 years) with assigned care levels have an increased risk of losing teeth and a higher prevalence of carious lesions than seniors without the need of nursing care [25].

With regard to these aspects, the current study aimed to investigate the social support and the oral health-related quality of life (OHRQoL) of older seniors in Leipzig, Germany, during the COVID-19 pandemic. The working hypotheses suggest no differences in the OHRQoL and the self-perceived social support of older seniors with and without an assigned care level.

## 2. Materials and Methods

### 2.1. Survey

For this cross-sectional study, a questionnaire with three parts was developed. In the first section, demographic characteristics of the participants were collected, such as age, sex, COVID-19 vaccination status (at least one vaccination shot), and assigned care level. The second part of the survey focused on patients’ behavior regarding dental appointments and their ability of finding, understanding, and using COVID-19-related information about health. The results of this part of the survey will be described elsewhere. The third section comprised an adapted German version of the ENRICHD Social Support Inventory (ESSI-D [26]) and the German version of the Oral Health Impact Profile (OHIP-14 [27,28]). Developed for the evaluation of self-perceived social support, the ESSI-D comprises five questions, addressing patients’ support with making decisions, solving problems, or showing love and affection. Answers to each item are presented as a Likert scale: none of the time (1), a little of the time (2), some of the time (3), most of the time (4), or all of the time (5), with higher scores representing a better social support. In accordance with the English version of the ESSI [26], “low social support” was defined with a threshold of ≤18 points, including a minimum of two items rated ≤3. In 2004, approximately 24.1% of the German population were identified as having low social support [14].

As a reliable and valid tool for the assessment of OHRQoL, the OHIP-14 consists of 14 standardized items. Each question can be answered using a Likert-like scale, whether or not the problem has never occurred (0), hardly never (1), occasionally (2), fairly often (3), or very often (4) within the previous month. Thus, lower scores correlate with higher OHRQoL. Of the 14 questions, two each can be divided into seven domains representing functional limitation, physical pain, psychological discomfort, physical disability, psychological disability, social disability, and handicap. A difference of two between sum scores of the OHIP-14 represents a “minimal important difference” (MID) and is, therefore, regarded as a threshold for clinical relevance [29]. As mean OHIP-14 scores of the general German population have not yet been described in the literature, mean values observed in the Swedish population (4.3 OHIP-14 points) were used as a European reference [30].

For the enrollment of participants, the survey was sent to all patients of the Dental Clinic of the University of Leipzig who had utilized any kind of outpatient dental service since October 2019, regardless of whether they additionally receive dental care at home or at other dental practices, and who were 75, 80 or 85 years old (±1 year if birthday and survey period overlapped). Being referred to as “older seniors” according to the Fifth German Oral Health study [25], these three age groups were supposed to give a good overview of the opinion of the group of older seniors. An enclosed information sheet provided background information on the purpose of the study and data protection; a stamped and addressed return envelope was added to the documents. All surveys were dispatched at the end of February 2021 and participants were asked to voluntarily return completed questionnaires anonymously by 31 March 2021. Replies after 14 April 2021 were not taken into account; no reminders were sent. The local ethical committee of Leipzig University reviewed and approved the concept and the questionnaires (005/21-ek).

### 2.2. Survey Pretesting

A pretesting of the questionnaires was performed in December 2020. Using the think-aloud strategy, thirteen subjects (mean age 81 years, 38% male) who never had dental treatment at the University of Leipzig before were asked to complete the survey in the presence of one of the authors and express their thoughts aloud. Afterwards, the participants were asked to return the survey according to the instructions in the enclosed information sheet. The aim of the pretesting was to point out parts of the survey that might lead to misunderstandings and to test its practicability.

### 2.3. Statistical Analysis

To perform statistical analyses (IBM SPSS 27, IBM, Armonk, NY, USA), frequencies were determined. For evaluation of the participants’ social support, sum scores of participants with five ESSI-D items were calculated. Using the Chi-square test, differences in self-perceived low social support in regard to the assigned care level were identified and odds ratios (OR) and 95% confidence intervals (CI) were assessed. Counts and percentages of each ESSI-D-item were calculated. For OHIP-14 analyses, imputation was performed for a maximum of one missing item sum score. Since no normal distribution was identified (Shapiro–Wilk test: *p* ≤ 0.001), the total sum scores and seven OHIP-14 dimensions were tested for significance in dependence on the assigned care level using the Mann–Whitney test. The level of significance was set to *p* < 0.050. The correlation between social support and OHRQoL was determined with Pearson correlation. The Pearson correlation coefficient (*r*) was interpreted as weak (*r* = 0.2–0.5), moderate (*r* = 0.51–0.7), strong (*r* = 0.71–0.9), or very strong (*r* > 0.9) [31].

## 3. Results

### 3.1. Demographic Characteristics

During the 6-week period of the survey, a total of 439 patients replied (response rate 35.7%). Twelve datasets were excluded as respondents did not belong to the demanded age groups, were cognitively impaired, or refused to participate—resulting in a total number of 427 datasets that were included in the analysis. While sex of the participants was almost equally distributed, age groups were split up to nearly one-quarter of patients aged 75 years, approximately one-half of patients aged 80 years, and another quarter of patients aged 85 years. Participants with a high care level (CL 4–5) were hardly represented, as they are often too immobile to visit the dental clinic. Additional data on demographics are presented in Table 1.

### 3.2. Social Support

For evaluation of the participants’ social support, only surveys including valid answers to all five ESSI-D-items were considered. As 24 surveys were incomplete, the remaining 403 surveys were used for data analysis. The majority of participants (86.8%, *n* = 350) met the criteria of “high social support”, while the other patients (13.2%, *n* = 53) were categorized as patients with “low social support”. When analyzing low social support according to ESSI-D in regard to the existence of an assigned care level, no statistically significant differences were identified (low social support: CL_no_/CL_yes_ 12.1/17.4%; *p* = 0.196). A descriptive evaluation of the items for ESSI-D is presented in Table 2.

### 3.3. OHRQoL

Of the 427 participants, 26 were not included in data analysis due to missing information regarding OHIP-14-items (*n* = 23) and/or care level (*n* = 3). The mean OHIP score of the remaining 401 patients was 7.2 (Table 3) and the median was 4.0 points. Depending on the patients’ assigned care level, statistically significant differences were identified for total sum scores (CL_no_/CL_yes_ 6.43/10.12; *p* < 0.001) and six of the seven domains.

### 3.4. Correlation between Social Support and OHRQoL

When analyzing the correlation between ESSI-D sum scores and OHIP-14 sum scores, a weak positive correlation was identified for seniors with an assigned care level (*r* = 0.29, *p* = 0.010), whereas for seniors without a care level, no significant correlation was observed.

## 4. Discussion

The results of the investigation revealed a statistically significant difference in the perception of OHRQoL by older seniors depending on the assigned care level. In contrast, no differences were identified for both groups with regard to their self-perceived social support. Consequently, the working hypotheses of the current investigation were partially rejected.

According to the results of the ESSI-D, 13.2% of the participants stated that they had low social support, which represents smaller values than investigated in German seniors by Cordes and colleagues (24.1%) [14]. Considering that more than three-quarters of the participants of the current study (78.5%) did not have an assigned care level, it can be assumed that these patients were rather living at home than in a long-term care facility. Therefore, they might be more involved in family life or spending time with friends. Patients of the dental clinic of the University of Leipzig usually attend dental services independently. Thus, it may be assumed that even those who indicated a care level (21.5%) were not living in long-term care facilities but in a flat or house with a spouse or other family members offering social support in addition to care when needed. Due to restrictions and social distancing during the COVID-19 pandemic, contact with close friends and family members possibly intensified and improved the perception of social support. Moreover, younger and low-risk people oftentimes supported older or frail relatives by running their errands—especially in times when COVID-19 vaccines were not available—reducing older peoples’ chance of meeting other people. This minimization of their social circle might have led to an even closer and more intimate relationship to the few people within the circle, which might serve as an explanation for the high self-perceived social support identified at the time that the survey was conducted. In addition, social support might have been high as older seniors possibly took the initiative to contact close friends and relatives in order to talk about potential worries, their well-being, and everyday life in times of the COVID-19 pandemic. It is also possible that the use of digital media led to increased self-perceived social support [32], as older seniors had the chance to be included in family life in a safe and non-physical way by attending online video calls.

Analysis of the OHIP-14 data revealed a mean sum score of 7.2, which is higher than the Swedish reference value of 4.3 [30]. In addition, the median sum score of 4.0 is clinically relevantly higher than values described in the literature. According to Hassel and colleagues, a median score of 2.0 was identified for older Germans in 2010 [33]. As this investigation was performed prior to the outbreak of the coronavirus, the higher median scores identified in the current investigation might be explained by a negative impact of the COVID-19 pandemic on the participants’ perception of their subjective oral health status. One of the reasons might be restrictions, such as the postponement of elective treatments that extended treatment periods and prolonged time with temporary dentures and restorations. Due to the fact that seniors with an assigned care level often utilize dental examinations on a complaint-associated basis [25], they might present oral health conditions in a progressed stage, leading to a decrease in their self-perceived OHRQoL.

In the subjects of the current investigation, the mean OHIP-14 sum score was 10.1 points. This is relevantly higher than identified by an investigation published in 2021 that reported a mean OHIP-14 sum score of 5.7 for older Germans living in long-term care facilities or being supported by a mobile nursing service [34]. Yet, there is no information available on whether or not the data collection was conducted prior to the COVID-19 pandemic. Still, it seems surprising that previous participants living in long-term care facilities stated a better OHRQoL than the older seniors in Leipzig, probably living on their own—especially when considering that almost 80% of the participants of the current study were not assigned with a care level. In regard to the observations of the Fifth German Oral health study that reported higher tooth loss and an increased prevalence of carious lesions in older seniors requiring care [25], one would assume a higher OHIP-14 sum score in older seniors dependent on caregivers. One of the reasons for the positively self-perceived OHRQoL of the older Germans living in long-term care facilities might be the “paradox of subjective well-being”, stating that people are able to feel good despite an exposure to adverse conditions, e.g., unfavorable oral conditions [35]. When analyzing the seven OHIP-14 domains, older seniors with an assigned care level presented a lower OHRQoL. The lowest *p*-value was observed for the domain “social disability” that addressed the patients’ perceived difficulties with doing their usual jobs or their irritation with other people due to difficulties with their mouth, teeth, or dentures. One of the reasons for the negative perception might be that older adults with assigned care levels presented minor impairment of their independence or everyday competencies [22] during the COVID-19 pandemic. The same reason might apply to the “handicap”-domain that addressed patients’ satisfaction regarding life in general depending on problems they experienced with their teeth, mouth, or dentures. The only domain that did not reveal statistically significant differences with regard to the existence of a care level was “functional limitations”. In contrast, Hassel and colleagues identified that older seniors living in a long-term care facility mainly experienced “functional limitations” regarding their OHRQoL, while the problem of “social disability” was mentioned the least [36].

A weak positive correlation between a high social support and an increased self-perceived OHRQoL of seniors with an assigned care level was investigated. As a positive correlation between social support and health-related quality of life was already identified [10], it is not surprising that similar associations were identified for the OHRQoL. While 29.8% of seniors with an assigned care level usually require external help with oral hygiene measures, this applies to only 6.7% of same-aged adults without a care level [25]. Consequently, seniors with lower social support during the pandemic might not be able to sufficiently perform oral health measures or attend medical appointments, resulting in a decreased OHRQoL. Another reason might be the dependency on close friends or relatives concerning, e.g., transportation or completing documents dealing with medical history.

As a limitation of the study, it should be noted that the survey did not address information regarding family background, including living situation or marital status. As social support is lower in singles or individuals living alone [14], this might have affected the way the participants of the present study evaluated their self-perceived social support. Moreover, the results of the current study might have been affected by characteristics of the subjects, as they were not randomly selected from older adults in the community but from patients of a dental clinic. As another shortcoming of this study, it should be mentioned that no information was collected on whether patients had previously been infected with COVID-19. Older seniors isolated from friends and family due to a COVID-19 infection might have experienced lower social support than a healthy person who lived without restrictions. In addition, oral-related symptoms of a COVID-19 infection such as coughing or loss of taste might have negatively affected the OHRQoL of subjects with a previous infection. Moreover, it was not possible to identify whether scores of OHIP-14 and ESSI-D decreased or increased in the examined subjects as no pre-pandemic data were collected.

## 5. Conclusions

Despite the COVID-19 pandemic, the results of the current investigation reported a high self-perceived social support of older seniors in Leipzig, Germany. As self-perceived OHRQoL was more impaired than observed in reference subjects, the COVID-19 pandemic might have negatively influenced patients’ self-perceived OHRQoL. Moreover, older seniors with an assigned care level presented higher OHIP-14 sum scores, indicating an impaired OHRQoL with a positive correlation to low social support. Therefore, special attention should be given to the improvement of oral health conditions and social support of older people with increased frailty, especially when pandemic restrictions are given. This might include, e.g., better options for visits of a doctor at home, separated waiting rooms in dental clinics, support in receiving optimal measures such as state-of-the-art masks, disinfectant tools, vaccines.

## Figures and Tables

**Table 1:** Demographic characteristics of the participants; missing data due to incomplete questionnaires.

	Total	Count	Percentage
Overall	427		
Sex	427		
female		215	50.4
male		212	49.6
Age group	427		
75		119	27.9
80		200	46.8
85		108	25.3
Care level (CL)	423		
no CL		332	78.5
CL 1		18	4.3
CL 2		44	10.4
CL 3		27	6.4
CL 4		1	0.2
CL 5		1	0.2
Vaccination status	415		
no		278	67.0
yes		137	33.0

**Table 2:** Descriptive evaluation of ESSI-D-items; % = percentage, *n* = counts; N = 400, missing data due to incomplete ESSI-D questionnaires (*n* = 23) or missing information regarding existence of a care level (*n* = 4).

Item	Question	Categories of Answers				
		1 None of the Time	2 A little of the Time	3 Some of the Time	4 Most of the Time	5 All of the Time
		% (*n*)				
ESSI-D 1	Is there someone available to you who you can count on to listen when you need to talk?	0.8 (3)	2.5 (10)	6.3 (25)	34.3 (137)	56.3 (225)
ESSI-D 2	Is there someone available to give you good advice about a problem?	1.3 (5)	3.0 (12)	10.0 (40)	35.0 (142)	50.7 (203)
ESSI-D 3	Is there someone available to you who shows you love and affection?	1.3 (5)	4.3 (17)	6.0 (24)	21.3 (85)	67.3 (269)
ESSI-D 4	Can you count on anyone to provide you with emotional support, such as talking over problems or helping you make difficult decisions?	1.5 (6)	3.5 (14)	6.8 (24)	22.3 (89)	66.0 (264)
ESSI-D 5	Do you have as much contact as you would like with someone you feel close to, someone you can trust and confide in?	0.8 (3)	6.0 (24)	5.0 (20)	22.3 (89)	66.0 (264)

**Table 3:** Mean values and standard deviations of OHIP-14 domains and sum score; N = 401; Q1 = 25th percentile; Q3 = 75th percentile; significance of differences depending on absence/presence of care level (CL_no_/CL_yes_); missing data due to incomplete questionnaires.

Domain	Mean ± SD (Q1/Median/Q3) *n* = 404			CL_no_/CL_yes_ *p*-Value (Mann–Whitney-Test)
	Overall (*n* = 401)	CL_no_ (*n* = 317)	CL_yes_ (*n* = 84)	
Functional limitation	0.66 ± 1.30 (0/0/1)	0.58 ± 1.17 (0/0/1)	0.98 ± 1.66 (0/0/2)	0.068 (n.s.)
Physical pain	1.32 ± 1.65 (0/1/2)	1.23 ± 1.61 (0/1/2)	1.69 ± 1.77 (0/1/3)	0.018
Psychological discomfort	1.20 ± 1.69 (0/0/2)	1.13 ± 1.69 (0/0/2)	1.48 ± 1.64 (0/1/2)	0.015
Physical disability	0.68 ± 1.35 (0/0/1)	0.60 ± 1.30 (0/0/1)	0.98 ± 1.49 (0/0/2)	0.013
Psychological disability	1.09 ± 1.49 (0/0/2)	0.97 ± 1.36 (0/0/2)	1.52 ± 1.86 (0/1/3)	0.041
Social disability	1.07 ± 1.46 (0/0/2)	0.91 ± 1.29 (0/0/2)	1.69 ± 1.86 (0/1/3)	<0.001
Handicap	1.18 ± 1.54 (0/1/2)	1.02 ± 1.35 (0/0/2)	1.79 ± 1.99 (0/1/3)	0.001
OHIP-14 sum score	7.20 ± 8.25 (1/4/11)	6.43 ± 7.74 (1/4/10)	10.12 ± 9.41 (2/7/17)	<0.001

## Data Availability

The datasets used and analyzed during the current study are available from the corresponding author on reasonable request.
